# Resistance exercise training in older men reduces ATF4-activated and senescence-associated mRNAs in skeletal muscle

**DOI:** 10.1007/s11357-025-01564-2

**Published:** 2025-02-27

**Authors:** Zachary D. Von Ruff, Matthew J. Miller, Tatiana Moro, Paul T. Reidy, Scott M. Ebert, Elena Volpi, Christopher M. Adams, Blake B. Rasmussen

**Affiliations:** 1 University of Texas Medical Branch, Galveston, TX USA; 2 Division of Endocrinology, Diabetes, Metabolism and Nutrition, Mayo Clinic, Rochester, MN USA; 3 University of Iowa, Iowa City, IA USA; 4 Department of Biomedical Sciences, University of Padova, Padua, Italy; 5 Department of Kinesiology, Nutrition, and Health, Miami University, Oxford, OH USA; 6 Barshop Institute for Longevity & Aging Studies, University of Texas Health Science Center at San Antonio, 7703 Floyd Curl Drive, MC 7756, San Antonio, TX 78229 USA; 7 Department of Cellular & Integrative Physiology, University of Texas Health Science Center at San Antonio, 7703 Floyd Curl Drive, MC 7756, San Antonio, TX 78229 USA

**Keywords:** Aging, Skeletal muscle, Resistance exercise, ATF4, Senescence

## Abstract

Sarcopenia increases the risk of frailty, morbidity, and mortality in older adults. Resistance exercise training improves muscle size and function; however, the response to exercise training is variable in older adults. The objective of our study was to determine both the age-independent and age-dependent changes to the transcriptome following progressive resistance exercise training. Skeletal muscle biopsies were obtained before and after 12 weeks of resistance exercise training in 8 young (24 ± 3.3 years) and 10 older (72 ± 4.9 years) men. RNA was extracted from each biopsy and prepared for analysis via RNA sequencing. We performed differential mRNA expression, gene ontology, and gene set enrichment analyses. We report that when comparing post-training vs pre-training 226 mRNAs and 959 mRNAs were differentially expressed in the skeletal muscle of young and older men, respectively. Additionally, 94 mRNAs increased, and 17 mRNAs decreased in both young and old, indicating limited overlap in response to resistance exercise training. Furthermore, the differential gene expression was larger in older skeletal muscle. Finally, we report three novel findings: 1) resistance exercise training decreased the abundance of ATF4-activated and senescence-associated skeletal muscle mRNAs in older men; 2) resistance exercise-induced increases in lean mass correlate with increased mRNAs encoding mitochondrial proteins; and 3) increases in muscle strength following resistance exercise positively correlate with increased mRNAs involved in translation, rRNA processing, and polyamine metabolism. We conclude that resistance exercise training elicits a differential gene expression response in young and old skeletal muscle, including reduced ATF-4 activated and senescence-associated gene expression.

**Supplementary Information:**

The online version contains supplementary material available at 10.1007/s11357-025-01564-2.

## Introduction

Sarcopenia, characterized by the progressive loss of skeletal muscle mass, strength, and function with advancing age, stands as a significant burden affecting the elderly population worldwide [1, 2]. This age-related decline in muscle health diminishes physical performance and elevates the risk of frailty, morbidity, and mortality, thereby imposing substantial healthcare challenges [2, 3]. Currently, the only known intervention that improves muscle strength in the setting of age-related muscle loss is resistance exercise. Resistance exercise has been shown as a promising intervention capable of mitigating the detrimental effects of sarcopenia through increases in tendon strength, bone mineral density, muscle strength and power, and physical performance [4–6]. While the benefits of resistance exercise in mitigating age-related muscle decline are widely acknowledged, the intricate nuances of its impact on the transcriptome across different age groups remain unclear.

Resistance exercise is a potent stimulator of skeletal muscle protein synthesis and is an effective strategy for skeletal muscle hypertrophy. However, recent investigations suggest that the molecular pathways and key transcripts responsible for skeletal muscle adaptations following resistance exercise are likely age dependent [7, 8]. For example, a recent meta-analysis revealed that both young and older adults demonstrate similar capacity for hypertrophy following resistance exercise, pathways involved in immune signaling and ECM-mediated mechanotransduction were only observed in the young [7]. Moreover, inter-individual variability in response to resistance exercise training is well-documented, with some individuals exhibiting robust gains in muscle mass and strength ("high responders"), while others show minimal or no improvements ("non-responders") despite adherence to training [9, 10]. The mechanisms underlying this variability remain unclear but likely involve differences in transcriptomic regulation, epigenetic modifications, and protein turnover rates. Understanding these individual differences is crucial for optimizing resistance exercise interventions, particularly in aging populations where adaptive capacity may be further constrained. Characterizing the age-dependent effects on the transcriptome is vital in comprehending the underlying mechanisms driving muscle adaptation to resistance exercise across various life stages, thereby offering crucial insights for tailored interventions targeting different age-related muscle phenotypes.

Despite the established benefits of resistance exercise on aging skeletal muscle, there is a shortage of studies that investigate the unique transcriptomic signatures that may be the basis for age-dependent adaptations to resistance exercise. In this study, we compared the effects of a 12-week resistance exercise program on the transcriptome in both young and older men. Our goal was to determine both the age-independent and age-dependent changes to the transcriptome that occur following resistance exercise training. Furthermore, we used a novel approach to Gene Set Enrichment analysis to determine which gene sets and pathways are positively associated with important resistance training outcomes, including changes in lean mass and muscle strength.

## Methods and materials

### Ethical approval

The following studies were approved by the Institutional Review Board of UTMB and were in compliance with the Declaration of Helsinki. These trials are registered at www.clinicaltrials.gov as NCT02999802 and NCT01749189.

### Human protocols

Data was pooled from two previous resistance exercise studies at the University of Texas Medical Branch (UTMB). These studies were conducted non-consecutively and used mildly different exercise training protocols. However, both protocols utilized progressive overload, used the same exercise equipment, and were monitored by exercise physiologist. For a detailed description of each study please see the original manuscripts [11, 12]. Briefly, recreationally active young (*n* = 8; 24 ± 3.3 years) and older (*n* = 10; 72 ± 4.9 years) males not currently engaged in a structured resistance exercise program were recruited for these studies. All participants provided written informed consent, and all potential participants underwent medical screening at the UTMB’s Institute of Translational Science – Clinical Research Center (ITS-CRC) to ensure subjects were healthy with no pre-existing conditions. Following the screening, participants completed a series of preliminary visits consisting of a pre-training study day and 3 non-consecutive days of exercise familiarization. During the pre-training study day, baseline body composition was evaluated by dual-energy x-ray absorptiometry (DEXA; GE Lunar iDXA, GE Healthcare, Chicago, IL, USA). Isometric knee extension and flexion strength was assessed at 60° via dynamometry (Biodex; Biodex Medical Systems, Shirley, New York, USA). Following an overnight fast, a percutaneous muscle biopsy of the *vastus lateralis* were obtained using a 5-mm Bergström biopsy needle using aseptic technique under local anesthesia (2% lidocaine). Skeletal muscle cross-sectional area was assessed via immunohistochemical analysis before and after exercise training and has been described previously [13]. Following these preliminary visits, subjects began a 12-week structured resistance exercise program (Table S1). Upon completion of 12 weeks of training, body composition and muscle strength were reassessed as described earlier. Post-training muscle biopsies were collected 72 h after the last resistance exercise session.

#### Exercise training – older participants

Exercise sessions were completed non-consecutively three days per week and were supervised by a qualified exercise professional. Exercise sessions (~ 60 min) began with a 10-min treadmill walk for a warmup, followed by 10 resistance exercises that target a mixture of upper and lower body musculature, and concluded with 10 min of stretching cooldown. Exercise selection included dumbbell/barbell chest press, leg press, lat pulldown, leg extension, shoulder press, sitting leg curl, dumbbell arm curl, sitting calf raise, triceps extension, and crunches. Rest intervals were set at 60–75 s between sets and ~ 1–2 min between exercises. Exercise intensity was programmed at 3 sets of 15 repetitions at 60% 1-RM for each exercise. Intensity gradually increased to 3 sets of 10 repetitions at 70% of 1-RM. Participants performed exercises to failure, with loads adjusted incrementally as their ability to exceed prescribed repetitions improved. Throughout the training period, participants maintained their usual recreational physical activities but refrained from adding additional strength-training sessions.

#### Exercise training – young participants

Exercise sessions lasted 60–75 min and were conducted three times per week on non-consecutive days under the supervision of personal trainers. In week 1, participants completed 3 sessions at 60% 1-RM with 3 sets of 10 repetitions per exercise. During weeks 2–8, two sessions per week were carried out at 70% 1-RM, with 3 sets of 10 repetitions, with the final set performed to momentary muscular failure. To mitigate injury risk, an additional exercise session was conducted at 60% 1-RM, with 3 sets of 10 repetitions without reaching muscular failure. From weeks 9–12, two sessions per week were conducted at 80% 1-RM, with 4 sets of 8 repetitions taken to momentary muscular failure, while a third session continued at 60% 1-RM. Exercise selection included chest presses, leg presses, curls, extensions, seated pull-downs and rows, calf raises, and abdominal exercises. Participants rested for 1–2 min between exercises and sets. Participants were allowed to maintain their recreational physical activity but instructed not to do any other strength training outside of the study.

### Skeletal muscle mRNA profiling

Total RNA was isolated from ~ 25 mg of skeletal muscle biopsy sample using a guanidinium thiocyanate-phenol–chloroform based method [14]. A detailed explanation of the original protocol can be found elsewhere [15]. The resulting RNA pellet was dissolved in DEPC-treated water and RNA concentration and purity were tested using a spectrophotometer (NanoDrop 2000; Thermo Scientific, Waltham, Massachusetts). Following RNA extraction, samples were sent to the University of Texas Medical Branch’s Next Generation Sequencing and Bioinformatics Core for library preparation and sequencing. Libraries were prepared with NEBNext Ultra II RNA (New England BioLabs, Ipswich, MA), pooled and sequenced on an Illumina NextSeq 550 High-output flow cell with the single-end 75 base protocol.

### Differential mRNA expression, gene ontology, and gene set enrichment analyses

Sequence reads were aligned to the *H. sapiens* reference genome hg38 using the Spliced Transcript Alignment to a Reference (STAR) software version 2.7.1a [16]. FastQC was used for quality control. The number of RNA-sequencing reads uniquely mapped to annotated genes was determined using FeatureCounts [17]. Normalization and differential gene expression analysis was performed using the DESeq2 1.40.2 software package [18]. Transcripts with a *p*-value < 0.05 were considered significantly differentially expressed, and corresponding Benjamin-Hochberg adjusted p-values (FDR) are also presented in Table S2. Differentially expressed genes were then subjected to Gene Ontology (GO) analysis using clusterProfiler and the enrichplot package in R [19, 20]. Transcripts were pre-ranked according to log2 fold-change (log2FC), and Gene Set Enrichment Analysis (GSEA) was performed using the Reactome gene sets database [21]. Significantly enriched gene sets were identified as those with an FDR < 0.05.

### Correlation analyses

To determine the relationship between changes in skeletal muscle mRNA expression and muscle size and strength, we first calculated the participant-specific log2FC in the abundance of each mRNA following exercise training using DESeq2 normalized read counts. Next, for each participant, we calculated the delta (post–pre) of each muscle phenotype (lean mass, leg extension strength, leg flexion strength, and CSA of Type II fibers). Using linear regression, a Pearson’s correlation coefficient (r) was calculated between the log2FC of each gene and the delta of each muscle phenotype (Table S5). Positive correlation between the change in mRNA expression and muscle size/strength are indicated by r values > 0, whereas r values < 0 indicate a negative correlation. To determine which gene sets are most correlated with changes in muscle phenotypes, transcripts were pre-ranked by r value for each muscle phenotype (Fig. 4A,Table S6) and subjected to GSEA analysis using the Reactome gene sets database [21].

### Statistics

Subject characteristic and muscle phenotype data are presented as mean ± SD. Differences in continuous variables at baseline and after resistance training were assessed via a paired t-test. Inter-group differences at baseline were assessed determined by an unpaired t-test. Comparisons were considered statistically significant at *P* < 0.05. All statistical analyses were performed using R 4.3.1 (www.r-project.org) or GraphPad Prism 10.1.1 software (GraphPad Software Inc.) as described in materials and methods.

## Results

### Resistance exercise training is associated with increased muscle size, strength and transcriptomic changes

**Fig. 1:**
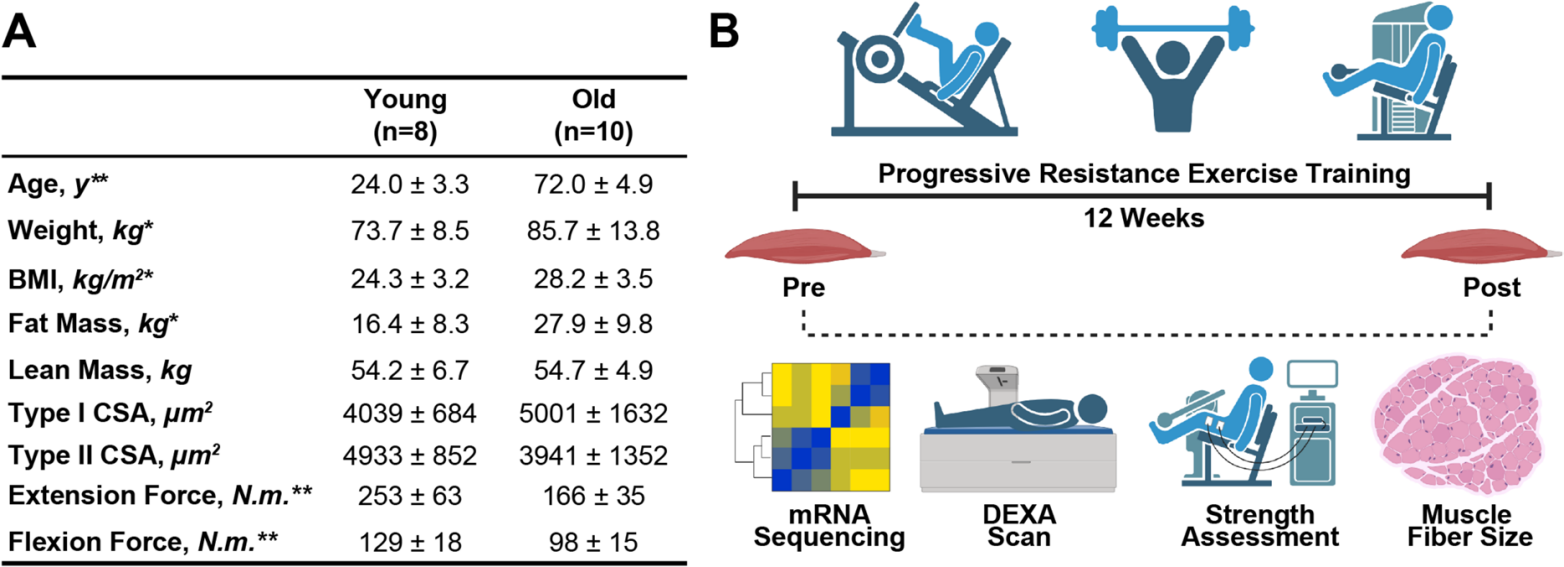
Progressive resistance exercise training study design. Human subjects who were 19- to 29-years-old (young) or 65- to 81-years-old (old) volunteered for a study in which body composition, leg strength, muscle fiber cross-sectional area (CSA), and muscle mRNA expression were analyzed before and after a 12-week resistance exercise training regimen. **A** Baseline subject characteristics. **B** Experimental overview. Values are means ± SD. BMI, body mass index. *p < 0.05, **p < 0.01

**Fig. 2:**
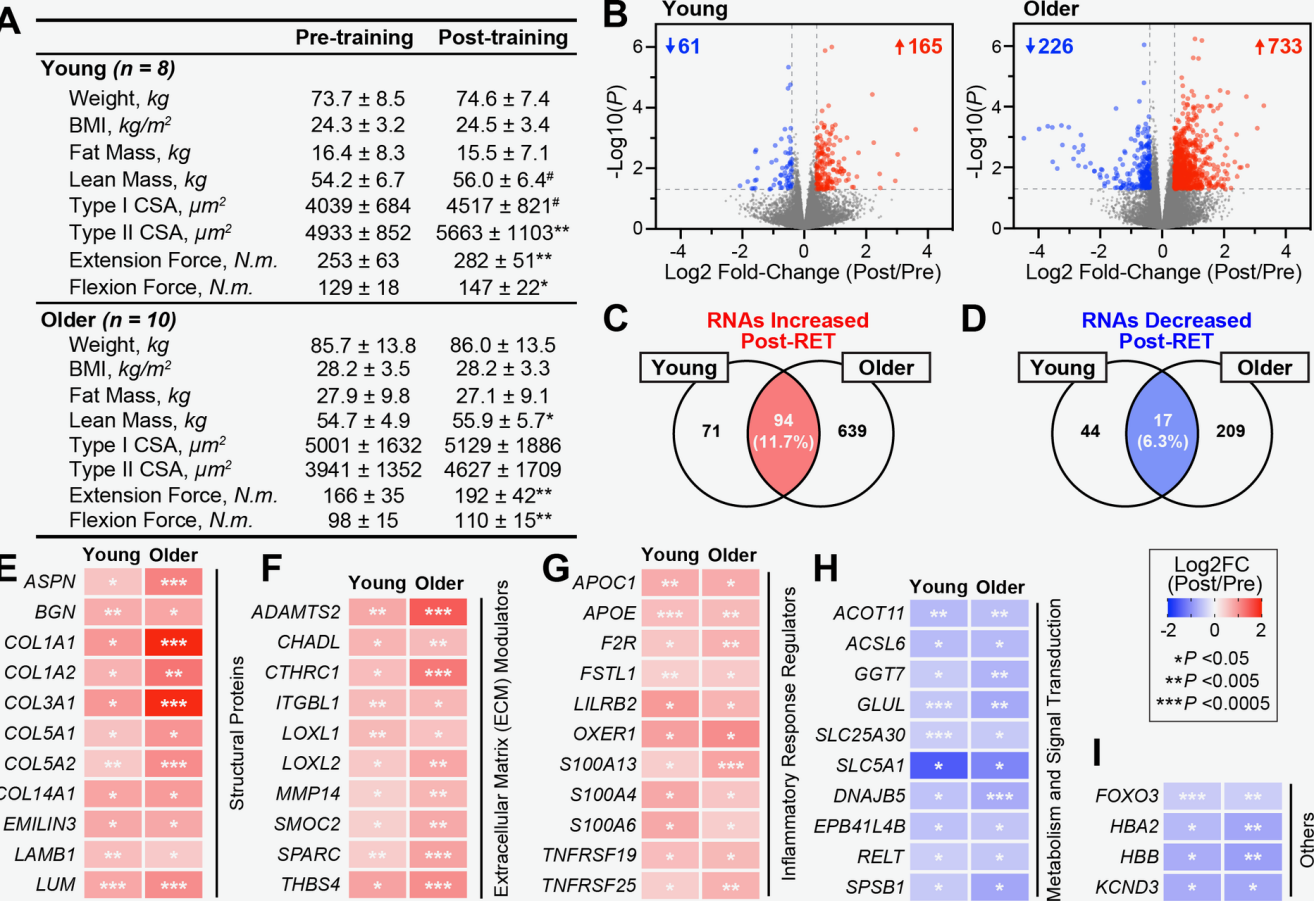
Changes in muscle mRNA expression post-resistance exercise training. **A** Study subject body composition, leg strength, and muscle fiber CSA after 12-weeks of resistance exercise training. #*p* < 0.1, *p < 0.05, **p < 0.01. **B** (C-D) Skeletal muscle RNAs that were significantly (*P* < 0.05; |L2FC|> 0.4) increased **C** and decreased **D** after 12-weeks of resistance exercise training based on analysis of young, old, and all participants combined. (E-I) Commonly increased (E–G) and decreased (H-I) skeletal muscle RNAs between young and older participants grouped by functional category

At baseline, older study participants possessed greater body weight, body mass index (BMI), and whole-body fat mass than younger participants, as expected. There was also a minor (0.5 kg) yet statistically significant difference in whole-body lean mass as measured by DEXA. Furthermore, older participants exhibited decreased knee extension and flexion strength relative to the younger cohort (Fig. 1A**)**. These results align with widely recognized patterns of aging-related shifts in body composition and muscle strength [1, 22, 23]. All study participants completed 12 weeks of structured resistance exercise training, after which a quantitative assessment of skeletal muscle phenotypes, including muscle size, strength, and mRNA expression, was performed (Fig. 1B). Following 12 weeks of progressive resistance training, both age groups exhibited significant increases in muscle strength as measured by isometric leg extension and flexion force. Increases in type II muscle fiber cross-sectional area (CSA) were observed in both groups following resistance exercise training, however, statistical significance was only observed in the younger group (Fig. 2A). Further, resistance exercise training led to significant increases in whole-body lean mass in the older participants, further indicating overall positive adaptations.. The robust increase in muscle strength following training in both cohorts, and its blunted effect on muscle size in older participants, is consistent with the concept of anabolic resistance in older individuals as well as observations in previous studies employing similar methodology [24–27]. To gain insight into the molecular changes associated with the physiological adaptations in muscle size and strength following resistance training, we performed RNA sequencing on pre- and post-training muscle biopsies from all study participants. Given that FDR correction resulted in minimal significant findings, we have included unadjusted p-values to aid in identifying potential candidates for future investigation (Table S2). Resistance exercise training induced global changes in mRNA abundance that were more pronounced in older individuals than younger individuals. In the younger cohort, a total of 226 mRNAs were significantly different, 61 (27.0%) decreased, and 165 (73.0%) increased in skeletal muscle biopsies taken post-training compared to pre-training baseline. By contrast, 959 mRNAs were altered in the older cohort, 226 (23.6%) decreased, and 733 (76.4%) increased following training (Fig. 2B,Table S2). Taken together, these results indicate that 12 weeks of resistance exercise training increases muscle strength in both young and older individuals, increases type II muscle fiber size in young, healthy individuals, and changes in RNA expression that are especially pronounced in the muscles of older individuals.

### Resistance exercise training consistently alters the expression of a limited number of skeletal muscle mRNAs between young and older adults

We compared training-responsive transcripts between young and older individuals to identify skeletal muscle transcriptional responses to resistance exercise that were common across all participants. To minimize the likelihood of type 2 errors in our analysis, we utilized unadjusted p-values to differentiate transcriptional responses between conditions. Rigorous statistical analysis, including Benjamin-Hochberg, corrected p-values, is presented in Table S2. This analysis revealed that 94 (11.7% of total number increased) RNAs were significantly increased in both study groups following 12 weeks of resistance exercise training (Fig. 2C). Among muscle mRNAs which increased across both cohorts, a subset encoded structural proteins including type I (*COL1A1, COL1A2*), type III (*COL3A1*) and type V (*COL5A1, COL5A2*) collagen chains, Laminin Subunit Beta (*LAMB1*), the glycoprotein Elastin Microfibril Interfacer 3 (*EMILIN3*) and several proteoglycans (*LUM, ASPN, BGN*) (Fig. 2E). Interestingly, the proteoglycan Lumican (*LUM*) is an exercise-induced skeletal muscle-secreted protein that has been shown to protect against muscle loss in mice [28]. In addition to these structural components, the expression of mRNAs encoding a wide range of extracellular matrix (ECM) modulatory proteins were increased post-training. This included Lysyl Oxidase-Like proteins (*LOXL1, LOXL2*) involved in collagen crosslinking and ECM stabilization [29], tissue regeneration-associated proteins such as Integrin-β-Like 1(ITGBL1) and Thrombospondin-4 (*THBS4*) [30–32], as well as Matrix Metallopeptidase 14 (*MMP14*), which has been directly implicated in skeletal muscle repair following hypertrophic exercise stimulus [33, 34]. Post-exercise training, muscles also showed an increase in mRNA encoding the Osteonectin (*SPARC*), a myokine with roles in muscle regeneration and function, whose downregulation with aging is associated with muscle atrophy [35–38] (Fig. 2F**)**. The ECM is an important mediator of muscle function, and age-related changes in the muscle ECM have been linked to negative outcomes, including the loss of muscle strength [39–43]. Resistance exercise training also led to an increase in skeletal muscle mRNAs encoding regulators of inflammatory responses across both young and older study participants, which included apolipoproteins (*APOC1, APOE*) [44–46], S100 calcium-binding proteins (*S100A13, S100A4, S100A6*) [47, 48] and members of the tumor necrosis factor receptor superfamily (*TNFRSF19, TNFRSF25*) [49–51] (Fig. 2F). Notably, Follistatin-Like Protein 1 (*FSTL1*) mRNA was also increased, which encodes a pro-inflammatory protein secreted by skeletal muscle in response to exercise, promoting angiogenesis and improved vascular endothelial function [52–55]. Taken together, these results identify an age-independent transcriptional response to resistance exercise that encompasses the induction of both structural and regulatory elements of the ECM as well as inflammatory mediators.

In parallel to upregulated transcripts, our analysis also revealed 17 (6.3% of total number decreased) mRNAs commonly decreased following 12 weeks of resistance exercise training in skeletal muscle from both young and older individuals, primarily involved in metabolism and signal transduction (Fig. 2D). Across both cohorts, exercise training reduced the levels of mRNAs encoding proteins with roles in lipid metabolism (*ACOT11, ACSL6*), amino acid metabolism (*GGT7, GLUL*), metabolic substrate transport mechanisms (*SLC25A30, SLC5A1*), and intracellular signaling pathways (*DNAJB5, EPB41L4B, RELT, SPSB1*) (Fig. 2H). Intriguingly, skeletal muscles also exhibited a consistent decrease in mRNAs encoding hemoglobin subunits (*HBA2, HBB*) as well as Forkhead Box O3 (*FOXO3*), a key transcription factor involved in the regulation of oxidative stress response and muscle atrophy [56–59] (Fig. 2I). The decreased FOXO3 mRNA suggests a potential adaptive response to resistance training, potentially reflecting reduced cellular stress and a shift towards muscle growth and repair. Collectively, these results highlight the limited overlap in the response of skeletal muscle to resistance exercise between younger and older individuals.

### Resistance exercise training results in age-dependent shifts in the skeletal muscle transcriptome

**Fig. 3:**
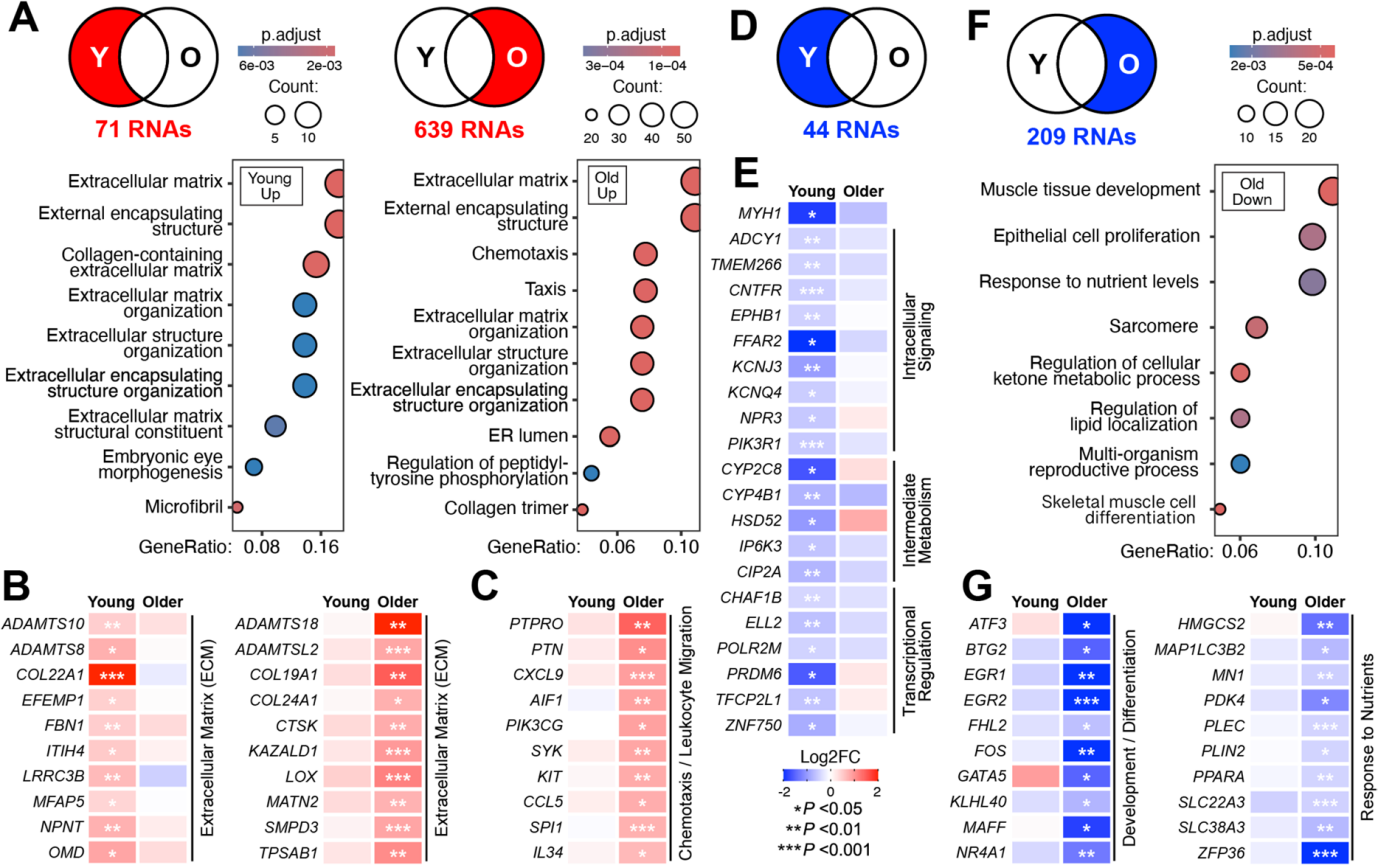
Age-dependent changes in muscle mRNA expression post-resistance exercise training. **A** Differentially expressed mRNAs that were uniquely upregulated in the young and old after 12-weeks of resistance exercise training (# *p* < 0.05; |L2FC|> 0.4). (B-C) Uniquely upregulated skeletal muscle RNAs of young **B** and older **C** participants grouped by functional category. **D** & **F** Differentially expressed mRNAs that were uniquely downregulated in the young and old after 12-weeks of resistance exercise training. **E** & **G** Uniquely downregulated skeletal muscle RNAs of young (E) and older (G) participants grouped by functional category

Age-dependent differences in the transcriptomic response to resistance exercise likely contribute to differential responses to resistance exercise observed between young and older study participants. Therefore, we sought to characterize mRNAs uniquely differentially expressed post-training for each age cohort. This analysis revealed that 639 mRNAs were specifically increased in the muscles of older participants, while 71 mRNAs were increased only in younger participants following 12 weeks of resistance exercise. As expected, several age-dependent resistance exercise-sensitive skeletal muscle mRNAs encode key regulators of muscle growth. For example, in older adults specifically, training increased skeletal muscle expression of Follistatin (*FST*) and Insulin-like growth factor-1 (*IGF1*) (Table S2). To systematically assess the age-dependent transcriptional response to exercise, we utilized gene ontology enrichment analysis. This analysis revealed strong upregulation of mRNAs with roles in the ECM functions in both cohorts, as was observed for commonly increased RNAs (Figs. 2D, E, 3A,Table S3). These results are not surprising as the skeletal muscle ECM is highly adaptable, and remodeling of the ECM is a well-established physiological response to resistance exercise [60–63]. Nonetheless, these data indicated differential transcriptional regulation of adaptive ECM responses between young and older subjects. Investigation of the ECM-related mRNAs most strongly affected between young and old subjects, three days following the last bout of resistance exercise, revealed distinct age-dependent transcriptional patterns. Younger muscles displayed unique upregulation of mRNAs known to play key roles in maintaining ECM structure and function, including Collagen Type XXII Alpha 1 Chain (*COL22A1*) [64], Fibulin-3 (*EFEMP1*) [65] and Fibrillin-1 (*FBN1*) [66, 67]. Specifically, *COL22A1*is related to the myotendinous junction, which facilitates the transmission of mechanical forces from the muscle fibers to the tendon [68]. By contrast, older muscles exhibited significant increases in multiple mRNAs encoding ECM-associated regulators of muscle regeneration that were not observed in muscles from younger participants, including Lysyl Oxidase (*LOX*) [69], Matrilin-2 (*MATN2*) [70] and Sphingomyelin Phosphodiesterase 3 (*SMPD3*) [71] (Fig. 3B). Furthermore, the muscles of older individuals experienced upregulation of transcripts involved in chemotaxis and leukocyte migration following training, which did not occur in younger muscles (Fig. 3A, C). Chemotaxis plays a crucial role in skeletal muscle repair following resistance exercise, primarily through the recruitment of immune and adult stem cells to the site of muscle damage [72, 73]. Taken together, these results may reflect a more pronounced and prolonged muscle repair and regeneration process post-resistance exercise in skeletal muscles of older adults.

Further analysis of differentially regulated exercise-responsive transcripts between cohorts revealed 209 and 44 mRNAs uniquely decreased in the muscles of older and younger participants, respectively, following resistance exercise training. In young individuals, 44 mRNAs were downregulated in response to training, which were not significantly enriched for any functional categories (Fig. 3D). These transcripts, decreased specifically in the younger cohort, encoded proteins involved in intracellular signaling, intermediate metabolism, and transcriptional regulation, without clear thematic convergence or a unified regulatory theme (Fig. 3E). On the other hand, following resistance training, muscles from older individuals exhibited repression of transcripts involved in muscle cell development and differentiation as well as the cellular response to low nutrient levels (Fig. 3F,Table S3). The repression of these pathways was particularly driven by a reduced abundance of mRNAs transcribed from early stress response genes. These immediate early genes typically undergo rapid and transient activation following muscle stress and injury and include *ATF3*, *EGR1*, *EGR2*, *MAFF, PPARA* and *NR4A1* [74–77] (Fig. 3E). Specific reductions in the expression of these transcripts in older individuals suggest a shift in the molecular stress response to resistance exercise in aging, possibly reflecting reduced efficiency of muscle repair in older individuals.

### Resistance exercise training decreases the abundance of ATF4-activated and senescence-associated skeletal muscle mRNAs in older adults

**Fig. 4:**
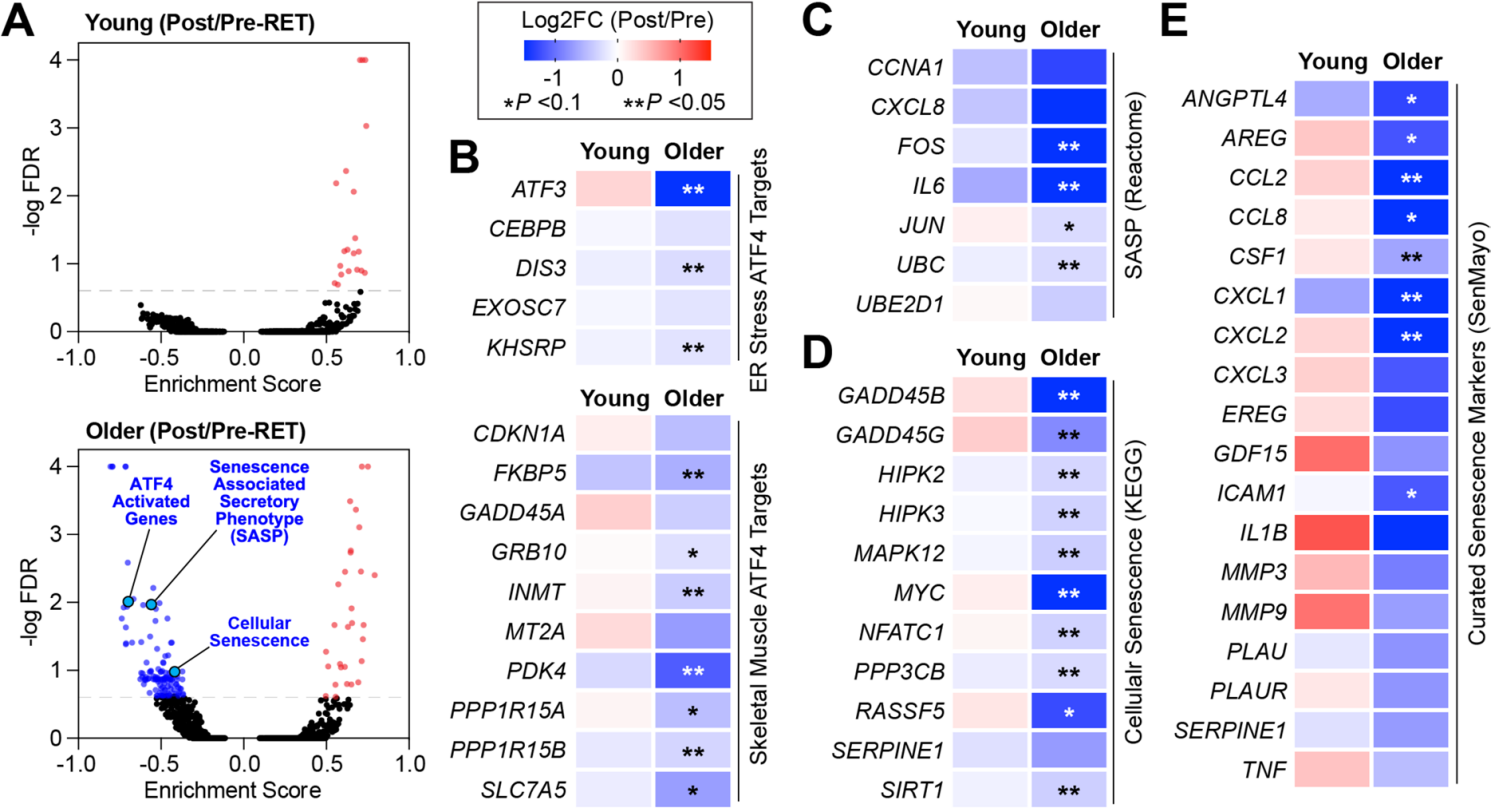
Resistance exercise downregulates transcripts involved in skeletal muscle aging in older participants. Gene set enrichment analysis (GSEA) of RNA-seq data was used to identify pathways that were altered following resistance exercise (FDR < 0.05). **A** Volcano plots of Reactome gene sets that were enriched and repressed in young and older participants respectively. (**B-E**) Individual transcripts that were downregulated in older participants grouped by functional category. *p < 0.1, **p ≤ 0.05

To further investigate the age-dependent effects of resistance exercise on the skeletal muscle transcriptome, we performed gene set enrichment analysis (GSEA) of global alterations in mRNA abundance. Like the effects on individual mRNA transcript abundance (Fig. 2B), this analysis identified many more differentially enriched gene sets in muscles from older adults, compared to young participants, following resistance exercise training (Fig. 4A,Table S4). Consistent with pathway analyses of differentially expressed transcripts, positively enriched gene sets in the older cohort were primarily involved in ECM structure and function. Interestingly, gene sets uniquely downregulated in muscles from older individuals post-training contained Activating transcription factor 4 (ATF4) activated genes in response to ER stress, as well as genes involved in cellular senescence and the senescence-associated secretory phenotype (SASP). During normal aging, ATF4 activity in skeletal muscle promotes the loss of muscle mass and strength [78–80]. Furthermore, cellular senescence and the SASP have emerged as a hallmark of multiple aging-related pathologies, including sarcopenia [81, 82]. ATF4 protein levels are primarily regulated at the translational level rather than through changes in mRNA abundance [83]. Consistent with this, we did not observe a difference in ATF4 mRNA levels following the resistance exercise training program (Log2FC = −0.08, *p* = 0.28, FDR > 0.99; Table S2). Furthermore, the half-life of ATF4 protein is less than one hour under non-stressed conditions, making direct quantification via western blotting or mass spectrometry particularly challenging [84]. Given these limitations, we assessed the expression of several well-established ATF4 target genes, such as GADD45A, as surrogate markers of ATF4 activity. Indeed, the abundance of numerous ATF4-dependent mRNAs was decreased specifically in muscles from older participants post-exercise training including *GADD45A*, *GRB10*, and PPP1R15A/GADD34 [80, 85] (Fig. 4B). Because of our previous work on ATF4 in muscle, we wanted to evaluate the relationship between skeletal muscle transcriptional responses to resistance exercise training and ATF4-dependent gene expression, we utilized skeletal muscle RNA-sequencing data from muscle-specific ATF4 knockout mice [80]. These mice, which are resistant to the typical reductions in skeletal muscle mass and strength that occur with normal aging, provide a pertinent model for evaluating the molecular changes associated with muscle loss in older adults. Specifically, we compared aging-induced ATF4-dependent mouse mRNAs against human mRNAs that were decreased by resistance exercise training to identify common transcripts. This analysis revealed 27 ATF4-dependent aging-induced mRNAs that were decreased in older individuals following training. These transcripts included *MT1A* and *PHLPP1*, which encode proteins that promote muscle atrophy, weakness, and ER stress [86, 87]. Furthermore, we identified training-induced reductions in skeletal muscle ATF4-dependent aging-induced mRNAs encoding the inflammatory mediators *CCL2* and *THBS1* [88, 89]. These findings related to ATF4-dependent gene expression align with reduced skeletal muscle expression of transcripts encoding SASP components in older individuals after resistance exercise.

The resistance exercise-induced reduction in SASP factors in older individuals was driven, in part, by decreased abundance of mRNAs encoding regulators of inflammatory responses (*CXCL8, IL6*) and ubiquitin-mediated proteolysis (*UBC, UBE2D1*) (Fig. 4C). Reductions in the cellular senescence pathway included transcripts that encode molecular mediators of stress responses, including kinases (*HIPK2, HIPK3, MAPK12*), transcriptional regulators (*MYC, NFATC1*), and growth arrest and DNA-damage-inducible transcripts (*GADD45B, GADD45G*) (Fig. 4D). To further investigate the connection between transcriptomic changes in response to resistance exercise and signatures of cellular senescence, we utilized a recently developed gene panel of cellular senescence markers (SenMayo) [90]. This analysis revealed significant repression of numerous transcripts contained within this curated gene set, specifically in the muscles of older individuals following training. Muscles from older individuals particularly demonstrated reduced expression of mRNAs encoding growth factors (*ANGPTL4, AREG, CSF1, EREG, GDF15*) and cytokines (*CCL2, CCL8, CXCL1, CXCL2, CXCL3, IL1B, TNF*) (Fig. 4E). Importantly, several of the factors encoded by these senescence-associated mRNAs reduced by resistance exercise in older adults are increased by aging and have been directly associated with the loss of muscle mass and strength [91–95]. Collectively, these results suggest that, in older adults, resistance exercise training decreases transcriptional activation of two distinct pathways involved in age-related muscle pathologies: ATF4 signaling and cellular senescence.

### Increased lean mass positively correlates with elevations in mRNAs encoding mitochondrial proteins

**Fig. 5:**
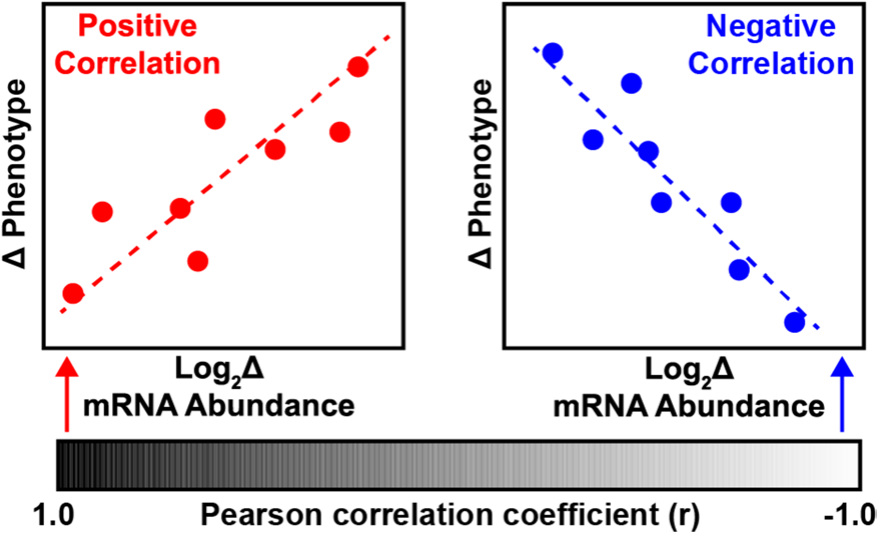
Schematic representation of strategy used for identification of mRNAs correlated with human muscle phenotypes

**Fig. 6:**
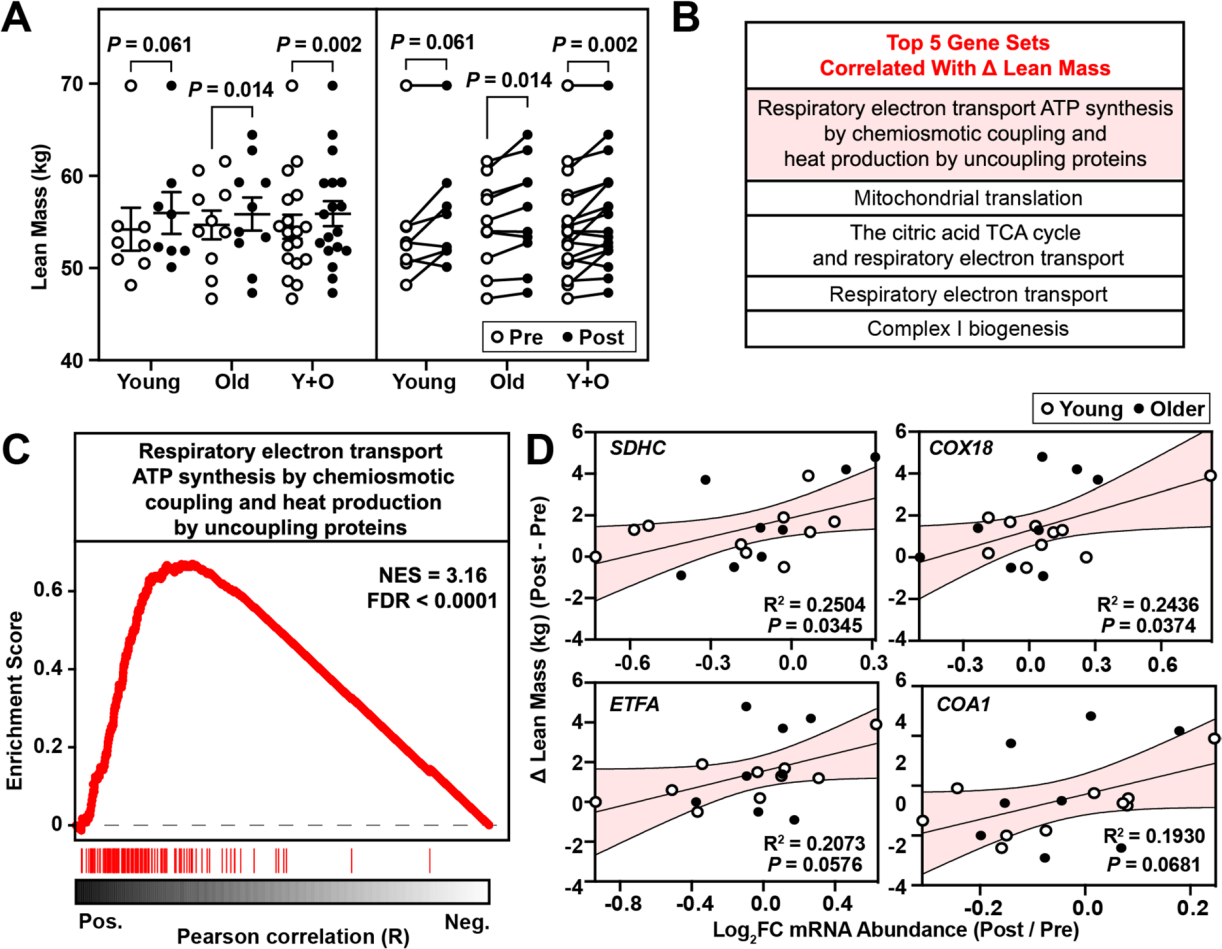
Genes sets positively correlated with increases in lean mass post-resistance exercise training. **A** Lean mass for each participant before (pre) and after (post) a 12-week progressive resistance exercise intervention. **B** GSEA was used to rank Reactome pathways based on their positive correlation with change in lean mass. **C** GSEA curve for the top positively associated Reactome pathway with changes in lean mass and the association of mRNA expression for key transcripts within those gene sets **D**

The skeletal muscle response to resistance exercise training can be highly variable among individuals [95]. Traditional analyses of changes in muscle size, strength, and molecular pathways induced by resistance training often group subjects into pre- and post-exercise categories, potentially masking the subtleties of individual responses. Recognizing the heterogeneity inherent in human responses to exercise, we next employed a subject-specific analytical approach to gain a more precise understanding of the molecular mechanisms that govern skeletal muscle response to exercise, described in detail in the methods section (Fig. 5). On average, whole body lean mass was increased following 12 weeks of resistance exercise training in young (*p* = 0.061) and older (*p* = 0.014) adults as well as all study participants combined (*p* = 0.002) (Fig. 6A). Despite the uniform training protocol, participants exhibited a substantial range in their responses, with changes in lean mass varying from −0.9 – 4.8 kg across all individuals involved in the study (Fig. 6B). Therefore, we hypothesized that the variability in resistance training responses is related to changes in the levels of specific skeletal muscle mRNAs. Consistent with this hypothesis, subject-specific correlation analysis revealed that the most strongly associated Reactome pathways corresponding to the changes in lean mass were all connected to mitochondrial function (Fig. 6C,Table S6). The effect of exercise on mitochondrial function varies greatly depending on exercise modality, intensity, and training status [96–99]. Furthermore, decreased mitochondrial function marked by lower mitochondrial protein synthesis rates, reduced mitochondrial enzymatic activity, and lower oxidative capacity are associated with skeletal muscle aging [100, 101]. Traditionally, aerobic exercise has been shown to be the more effective modality for improving mitochondrial function, however, recent evidence has been presented that demonstrates resistance exercise increases mitochondrial fractional synthesis rates and abundance of mitochondrial proteins [98]. We further investigated which mRNAs are most positively correlated with increases in lean mass in the highest enriched gene set. This analysis revealed that increases in lean mass are positively correlated with the increased expression of mRNAs known to play pivotal roles in mitochondrial function, including electron transport chain (*SDHC, COX18*) [102, 103], fatty acid and amino acid metabolism (ETFA) [104], and oxidative phosphorylation (*COA1*) [105] (Fig. 6D). These findings underscore the critical role of mitochondrial dynamics in modulating skeletal muscle adaptation to resistance training [106–108].

### Increased leg strength positively correlates with elevations in mRNAs involved in translation, rRNA processing, and polyamine metabolism

**Fig. 7:**
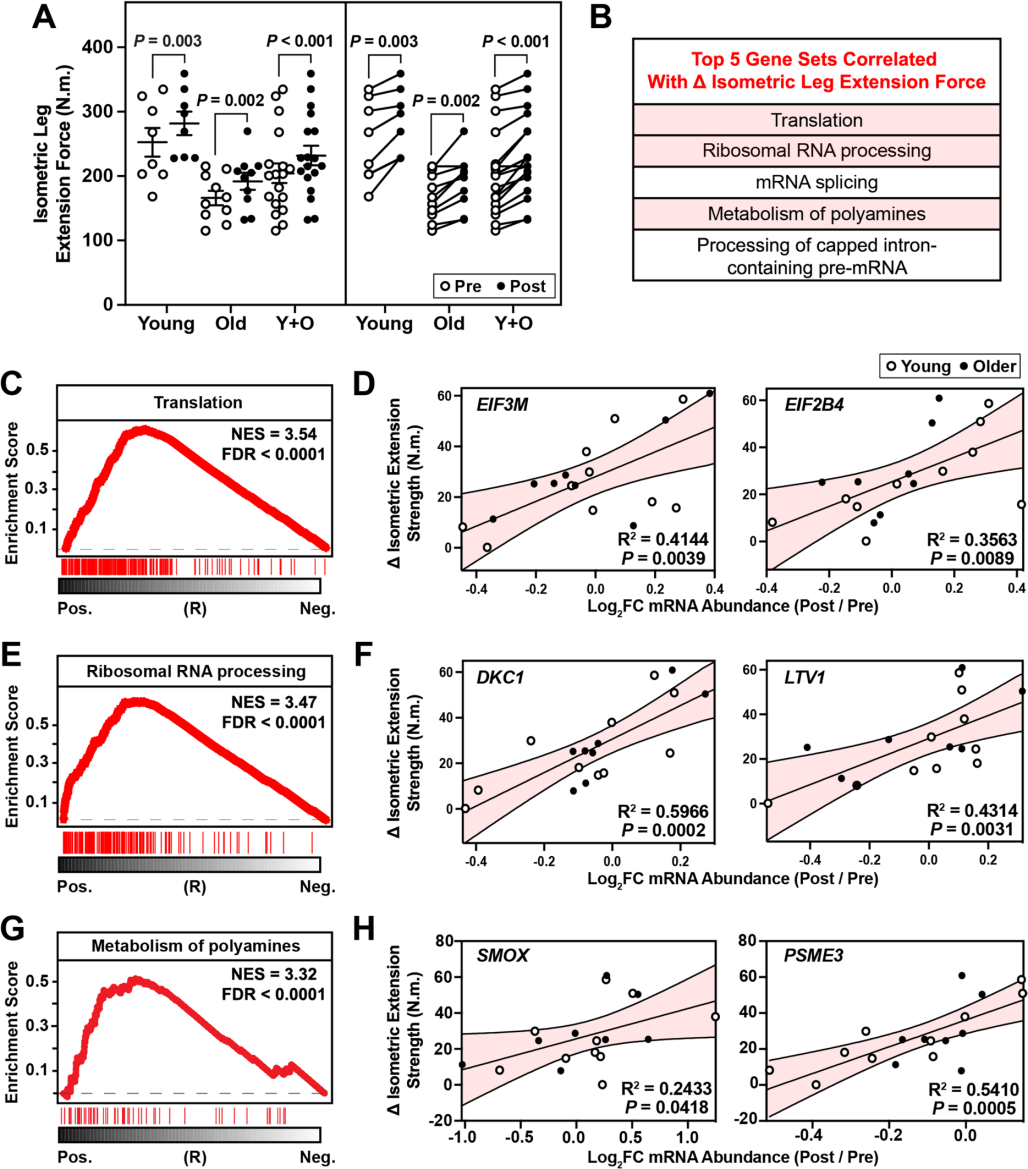
Gene sets positively correlated with increases in knee extension isometric strength post-resistance exercise. **A** Isometric knee extension strength for each participant before (pre) and after (post) a 12-week progressive resistance exercise intervention. **B** GSEA was used to rank Reactome pathways based on their positive correlation with change in lean mass. **C**, **E**, & **G** GSEA curves for the top positively associated Reactome pathways with changes in isometric knee extension strength and the association of mRNA expression for key transcripts within those gene sets **D**, **F**, & **H**

Repeated bouts of resistance exercise are an effective strategy to increase strength, stability, force production, and slow the progression of sarcopenia [109–111]. Because this study utilized RNA-seq data from vastus lateralis muscles, changes in leg extension strength are most relevant to the transcriptional changes observed following exercise. On average, increases in isometric knee extension strength were observed following 12 weeks of resistance exercise training in young (*p* = 0.003), old (*p* = 0.002), and combined groups (*p* < 0.001) (Fig. 7A). Inter-subject variability in changes of knee extension strength post-training ranged from 0.15 – 60.9 N.m. across all study participants (Fig. 7B). We utilized the same analytical approach for subject-specific correlation outlined in Fig. 5 to interrogate the transcriptomic changes underlying this variability in response to strength training, we investigated Reactome pathways correlated with increases in strength (Table S6**)**. This analysis identified that pathways involved in translation, ribosomal RNA processing, and polyamine metabolism were among the top five pathways positively correlated with increases in leg strength. Translation is essential for the response of skeletal muscle to exercise and plays a key roles in muscle repair [112], protein turnover [113], and mitochondrial biogenesis [114] (Fig. 7C). Within the translation gene set, increased mRNA expression of eukaryotic translation initiation factors such as *EIF3M* and *EIF2B4* were most positively associated with increases in leg strength (Fig. 7D). Eukaryotic initiation factor complexes play essential roles in the regulation of muscle protein synthesis, potentially contributing to improved muscle function [115, 116]. The second most positively correlated gene set with increases in leg strength was ribosomal RNA processing (Fig. 7E). Ribosomal RNA makes up over 85% of the total RNA pool in skeletal muscle and serves as a marker of translational capacity [117, 118]. While increases in ribosomal content post resistance exercise do not singularly predict muscle hypertrophy, they likely play a role in individualized responses to exercise, potentially influencing protein synthesis rates and adaptive outcomes in muscle development [119, 120]. Based on our analysis, changes in the levels of mRNAs encoding the ribosomal processing enzymes *DKC1* and *LTV1* specifically were most positively correlated transcripts with increases in leg strength (Fig. 7F). *DKC1* and *LTV1* have not been extensively studied in skeletal muscle, however, they have been shown to play a role in modification of ribosomal RNA pseudouridylation [121] and ribosomal biogenesis [122] respectively, suggesting their potential importance in regulating muscle protein synthesis and the adaptive hypertrophic response to resistance training. Interestingly, changes in mRNAs encoding enzymes involved polyamine metabolism were also strongly positively correlated with increases in muscle strength (Fig. 7G). The polyamine biosynthesis pathway products spermine and spermidine are important growth factors that play pivotal roles in cell growth, proliferation, and differentiation [123]. In the context of skeletal muscle, polyamine metabolism is negatively associated with conditions that cause muscle atrophy [124] and positively associated with muscle hypertrophy following resistance exercise [125]. Among mRNAs encoding enzymes involved in polyamine synthesis, increased *SMOX* and *PSME3* were among the most positively correlated with increases in leg strength (Fig. 7H). *SMOX* and its reaction product spermidine may contribute to muscle hypertrophy through increase autophagy and inhibition of apoptotic pathways [126, 127]. In summary, these data suggest that mRNA translation, rRNA processing, and polyamine metabolism are among the molecular pathways that most strongly contribute to improvements in skeletal muscle strength following resistance exercise.

## Discussion

In this study, we sought to characterize the effects of resistance exercise on the skeletal muscle transcriptome in both young and older adults. To this end, we utilized RNA-Seq data from human muscle biopsies from two independent cohorts of young and older adults before and after 12 weeks of progressive full-body resistance exercise. Our findings identify widespread age-independent and -dependent resistance exercise-induced changes to the skeletal muscle transcriptome. Notably, specifically in older adults, resistance training reduced the expression of key pathways related to skeletal muscle aging, which included mRNAs involved in cellular senescence and activated by ATF4-dependent stress signaling. Further investigations based on study subject-specific responses revealed that increases in lean mass were correlated with upregulation of pathways related to mitochondrial function, whereas increases in leg strength were positively associated with mRNAs linked to translation, rRNA processing, and polyamine metabolism.

The accumulation of senescent cells, which develop a senescence-associated secretory phenotype (SASP), is a hallmark of aging [90, 128]. The SASP consists of secreted factors, including interleukins, chemokines, proteases, and matrix remodeling proteins that negatively affect tissue function at a local and systemic level [129–131]. Skeletal muscles from older individuals exhibited a downregulation in senescence-associated gene sets, including the SASP, following 12 weeks of resistance training. Consistent with our findings, several previous studies have reported potential reductions in cellular senescence associated with resistance exercise. Senescent cells likely play a role in age-associated inflammation via the numerous sources of proinflammatory cytokines found within the SASP [132]. Compared to their inactive counterparts, physically active older individuals exhibit lower circulating levels of proinflammatory monocytes and TNF-alpha cytokine production. Furthermore, in physically inactive older adults, 12 weeks of combined aerobic and resistance exercise reduced TNF-alpha production and the proportion of proinflammatory monocytes, indicating the anti-inflammatory effects of exercise [133, 134]. Another recent study demonstrated that combined training reduced circulating markers of cellular senescence, including the expression of Cyclin Dependent Kinase Inhibitors p16 and p21 [135]. In rodents, aerobic exercise training has been shown to reduce the expression of senescence associated transcripts p53, p21, and IL-6 [136] in the liver and upregulate telomere stabilizing proteins in the heart [137]. In humans, duration of aerobic exercise (minutes per month) is inversely related to various blood markers of senescence including DNA damage, telomere dysfunction, and Cyclin Dependent Kinase Inhibitor 2A (*CDKN2A*) mRNA expression [138]. Interestingly, in our study, muscles from older individuals also exhibited decreased expression of ATF4 target genes following 12 weeks of resistance exercise. ATF4 is a stress-induced transcription factor that is both necessary and sufficient for the loss of muscle mass and strength that occurs during normal aging [80]. ATF4 activity in skeletal muscle, and the subsequent expression of downstream ATF4 target genes, is upregulated in response to a variety of conditions that induce skeletal muscle atrophy, including starvation, immobilization, and aging [139]. Previous data from our lab has demonstrated that the ATF4 target Gadd45a is upregulated in middle-aged adults following 7 days of disuse [140]. Taken together, these data suggest that resistance exercise may be an effective strategy to reverse some of the transcriptional changes associated with skeletal muscle aging in older adults.

Increases in muscle mass and strength are associated with improved quality of life and functional capacity [141]. Nonetheless, individuals respond variably to resistance exercise training, even within highly structured exercise programs [142]. While the benefits of exercise on quality of life are well documented, few studies have explored which gene expression patterns are most correlated with changes in important muscle phenotypes (i.e. lean mass, strength, muscle size) following resistance training. Using a novel approach to GSEA, we identified gene sets most strongly correlated with changes in lean mass following resistance exercise. This approach identified several gene sets related to skeletal muscle mitochondrial function, including the TCA cycle, mitochondrial translation, and complex I biogenesis, that were highly correlated with increases in lean mass following resistance training. Reductions in mitochondrial function occur with aging and are characterized by reduced mitochondrial biogenesis, enzymatic activity, expression of mitochondrial proteins, and mitochondrial DNA, and are associated with loss of skeletal muscle with aging [143, 144]. Exercise, particularly aerobic exercise, has been shown to increase mitochondrial content and function and increase mitochondrial biogenesis via peroxisome proliferator-activated receptor γ coactivator-1α (PGC-1α) expression [145, 146]. However, the effect of resistance exercise on mitochondrial function has shown mixed results. Several studies have shown that resistance training leads to mitochondrial dilution and reductions or no change in enzymatic activity of mitochondrial enzymes, including citrate synthase and succinate dehydrogenase [147, 148]. In contrast, previous data from our lab found that 12-weeks of resistance exercise training led to increased mitochondrial respiration and increased expression of NADH oxidase, a protein expression of complex 1 [149]. Similarly, resistance exercise alone has been shown to be more effective than combined training for increasing mitochondrial DNA in older adults [98]. Our results add to the growing literature that resistance exercise-induced increases in lean muscle mass are associated with improved mitochondrial function.

The importance of muscle strength in healthy aging is paramount, influencing physical resilience and reducing all-cause mortality [150]. In both young and older adults, 12 weeks of resistance exercise significantly increased knee extension and flexion isometric strength. Investigation of gene expression associated with subject-specific increases in leg strength revealed a strong positive correlation with mRNAs involved in translation, rRNA processing, and polyamine metabolism. Translation acts as a regulatory hub, enabling the synthesis of key myogenic proteins like myosin and actin that are crucial for muscle contraction and force generation during resistance exercise. The synthesis of muscle proteins is governed by the protein complex mechanistic target of rapamycin complex 1 (mTORC1) and is activated by various stimuli including nutrients, growth factors, and mechanical tension [151]. It is well documented that mTORC1 plays a crucial role in the adaptive response to resistance exercise, and its increased activity following resistance exercise contributes to skeletal muscle hypertrophy [152]. Our lab and others have reported strong associations between increased muscle protein synthesis rates and mTORC1 signaling following resistance exercise [153–155], indicating that mRNA translation plays a key role in the adaptive response to resistance exercise. Pre-rRNA processing plays a crucial role in ribosomal biogenesis through the modification and maturation of rRNA from its precursor form to its functional state within the ribosome [156, 157]. Notably, regulators of ribosomal biogenesis and ribosomal content have been shown to be attenuated following resistance exercise in aged animals and humans. For example, older individuals following 6-weeks of resistance exercise had reduced mTORC1 activity, reduced expression of transcripts involved in ribosomal biogenesis (i.e., c-MYC, TIF1a), and no change in ribosome content, ultimately resulting in a reduced hypertrophic response compared to young adults [158]. These data suggest that rRNA processing likely contributes to the individual response to resistance exercise. Interestingly, gene sets associated with polyamine metabolism were strongly correlated with increases in leg strength. Previous studies have suggested a role for polyamines in skeletal muscle homeostasis, with implications for both muscle hypertrophy and atrophy. For example, mechanical overload upregulates muscle Adenosylmethione decarboxylase 1 (Amd1) expression in mice [125]. Amd1 is a rate-limiting enzyme in the polyamine pathway that plays a crucial role in the biosynthesis of polyamines. Another important enzyme in the polyamine pathway, Spermine oxidase (SMOX), is highly expressed in skeletal muscle and plays an important role in the maintenance of muscle fiber size [124]. Several studies have demonstrated that SMOX expression is repressed during multiple conditions that promote muscle atrophy. By contrast, the overexpression of SMOX has been shown to reduce muscle atrophy and increase muscle fiber size [124, 159]. Spermidine, a catabolic product of SMOX, may also play a crucial role in muscle fiber size maintenance during aging. Previous studies have shown that spermidine supplementation alongside resistance exercise improves autophagy biomarkers (i.e., Beclin1, LC3-II/LC3-I ratio) and reduces markers of cell death (i.e., Bax, caspase-3) in muscle atrophy conditions [126, 127]. This data suggests that polyamine metabolism plays a crucial role in the adaptive response to resistance exercise in skeletal muscle.

Our study leverages several strengths that contribute to a more comprehensive understanding of the effects of resistance exercise on the skeletal muscle transcriptome. Notably, our analysis incorporates RNA-seq data from both young and older participants, allowing us to investigate the age-specific changes to the transcriptome induced by resistance exercise. Additionally, unlike many other studies that often combine resistance and aerobic training, our focus solely on resistance exercise enables a more specific examination of its effects on the transcriptome. Moreover, the novel GSEA-based approach to subject-specific changes in gene expression, lean mass and strength has enabled us to unveil pathways intricately associated with critical muscle phenotypes. This innovative method provides valuable insights into potential mechanisms underlying exercise-induced adaptations and serves as a framework for generating hypotheses to steer future investigations into targeted pathways influencing muscle health and performance across multiple age groups.

Nonetheless, certain limitations need to be considered before interpreting our findings. Although both participants underwent 12-weeks of resistance exercise training, the young cohort were prescribed higher volume and intensity, which might confound the results. Primarily, our study focused on mRNA levels and did not directly measure specific markers of muscle function (i.e., mitochondrial function, polyamine metabolism) which could offer more direct insights into the mechanistic changes induced by resistance exercise. Although we report changes in senescence-related gene sets and transcripts encoding proteins involved in the SASP, we did not observe significant changes in classic senescent cell-associated genes (i.e., CDKN1A, CDKN2A, TP53, STING1, or HMGB1). Additionally, changes in transcript quantity may also reflect a compensatory mechanism due to bottlenecking after transcriptional regulation, rather than a direct indicator of altered cellular function. Furthermore, our analysis does not directly analyze changes in senescence cell abundance in muscle tissues. Moreover, the analyses presented here inherently lack the capacity to establish causation between variables involved in the differential response of muscle to resistance exercise across individuals and age groups. As such, while our results identify intriguing associations, their physiological significance requires further validation. Specifically, mechanistic studies or interventional trials are warranted to determine the precise impact and direction of influence these pathways may have on muscle function and exercise-induced adaptations. Such work, including protein-level verification and single nuclei RNA-seq, will refine our current conclusions and guide subsequent investigations. Finally, our study was done in men only and future work should address sex-based differences in resistance exercise training responses in older men and women.

This study aimed to comprehensively investigate the impact of resistance exercise on the transcriptome across different age groups. By analyzing RNA-Seq data from muscle biopsies of both young and older adults after a 12-week resistance exercise program, we revealed age-specific alterations to the transcriptome. Notably, older adults exhibited reduced expression in pathways linked to skeletal muscle aging following resistance exercise training, including cellular senescence and ATF4 signaling. Additionally, we observed positive correlations between pathways associated with mitochondrial function and increases in lean mass, while pathways involving translation, rRNA processing, and polyamine metabolism were linked to enhanced leg strength. These findings underscore the differential effects of resistance exercise on the skeletal muscle transcriptome concerning age-related changes and functional outcomes in muscle size and strength. Further mechanistic studies will be needed to determine how resistance exercise training in older adults reduces senescence-associated gene expression in skeletal muscle.

## Supplementary Information

Below is the link to the electronic supplementary material.

Supplementary file1 (DOCX 16 KB)

Supplementary file2 (XLSX 3931 KB)

Supplementary file3 (XLSX 31 KB)

Supplementary file4 (XLSX 84 KB)

Supplementary file5 (XLSX 2289 KB)

Supplementary file6 (XLSX 76 KB)

Supplementary file7 (XLSX 243 KB)
