# Supplementary material for: Resistance exercise training in older men reduces ATF4-activated and senescence-associated mRNAs in skeletal muscle
Source: GeroScience. 2025 Feb 27;47(3):4601–22. doi: 10.1007/s11357-025-01564-2 (PMC12181168; doi:10.1007/s11357-025-01564-2)
Supplement: Supplementary file 1 — Supplementary file1 (DOCX 16 KB) [file 11357_2025_1564_MOESM1_ESM.docx]

**Supplemental Table 1: Exercise protocols for both young and older cohorts**

**Young Adults**

|  | **Week 1** | **Weeks 2-8** | **Weeks 9-12** |
| --- | --- | --- | --- |
| Frequency | 3/week | 3/week | 3/week |
| Intensity | 60% 1RM | 60% 1RM, one day 70% two days | 60% 1RM, one day 80% two days |
| Time | ~60-70 min | ~60-70 min | ~60-70 min |
| Type | RET | RET | RET |
| Volume | 3x10 | 3x10 to failure or 3x10 light | 4x8 to failure or 3x10 light |
| Progression | Strength was re-tested at 3, 6 and 9 weeks so as participants strength increased, absolute training loads could be adjusted to maintain a relative training intensity between 60-80% 1-RM. | | |

**Older Adults**

|  | **Weeks 1-4** | **Weeks 5-8** | **Weeks 9-12** |
| --- | --- | --- | --- |
| Frequency | 3/week | 3/week | 3/week |
| Intensity | 60% 1RM | 65% 1RM | 70% 1RM |
| Time | 60 min | 60 min | 60 min |
| Type | RET | RET | RET |
| Volume | 3x15 | 3x12 | 3x10 |
| Progression | 5% load increase when exceed prescribed repetitions | 5% load increase when exceed prescribed repetitions | 5% load increase when exceed prescribed repetitions |
